# *Cryptococcus neoformans* Overcomes Stress of Azole Drugs by Formation of Disomy in Specific Multiple Chromosomes

**DOI:** 10.1371/journal.ppat.1000848

**Published:** 2010-04-01

**Authors:** Edward Sionov, Hyeseung Lee, Yun C. Chang, Kyung J. Kwon-Chung

**Affiliations:** Molecular Microbiology Section, Laboratory of Clinical Infectious Diseases, National Institute of Allergy and Infectious Diseases, NIH, Bethesda, Maryland, United States of America; David Geffen School of Medicine at University of California Los Angeles, United States of America

## Abstract

*Cryptococcus neoformans* is a haploid environmental organism and the major cause of fungal meningoencephalitis in AIDS patients. Fluconazole (FLC), a triazole, is widely used for the maintenance therapy of cryptococcosis. Heteroresistance to FLC, an adaptive mode of azole resistance, was associated with FLC therapy failure cases but the mechanism underlying the resistance was unknown. We used comparative genome hybridization and quantitative real-time PCR in order to show that *C. neoformans* adapts to high concentrations of FLC by duplication of multiple chromosomes. Formation of disomic chromosomes in response to FLC stress was observed in both serotype A and D strains. Strains that adapted to FLC concentrations higher than their minimal inhibitory concentration (MIC) contained disomies of chromosome 1 and stepwise exposure to even higher drug concentrations induced additional duplications of several other specific chromosomes. The number of disomic chromosomes in each resistant strain directly correlated with the concentration of FLC tolerated by each strain. Upon removal of the drug pressure, strains that had adapted to high concentrations of FLC returned to their original level of susceptibility by initially losing the extra copy of chromosome 1 followed by loss of the extra copies of the remaining disomic chromosomes. The duplication of chromosome 1 was closely associated with two of its resident genes: *ERG11*, the target of FLC and *AFR1*, the major transporter of azoles in *C. neoformans*. This adaptive mechanism in *C. neoformans* may play an important role in FLC therapy failure of cryptococcosis leading to relapse during azole maintenance therapy.

## Author Summary

*Cryptococcus neoformans* is an environmental fungus that causes life threatening brain disease, primarily in AIDS patients. The disease is estimated to claim 700,000 lives annually world-wide but most heavily in Africa. Fluconazole (FLC), a fungistatic antifungal drug, is commonly used to treat patients for long term maintenance therapy. Recurrence of cryptococcosis in AIDS patients undergoing FLC maintenance therapy has been increasingly reported. Heteroresistance, an adaptive azole resistance, was associated with FLC therapy failure cases but the mechanism underlying the resistance was unknown. We previously described that *C. neoformans* strains are innately heteroresistant to FLC; each strain producing a fraction of subpopulation that can tolerate a high concentration of the drug. These resistant subpopulations revert to original phenotype during maintenance in drug free media. Various methods including cDNA microarrays, comparative genome hybridization and quantitative PCR have been applied to uncover the mechanism involved in the adaptation of *C. neoformans* to high concentrations of FLC and subsequent loss of resistance upon the removal of drug pressure. We discovered that *C. neoformans* adapts to high concentration of FLC by formation of disomy in multiple chromosomes. The removal of drug pressure results in a sequential loss of the extra chromosomal copies. It is likely that this novel mechanism of adaptation contributes to the failure of FLC therapy for cryptococcosis.

## Introduction

*Cryptococcus neoformans* is the most common cause of fungal meningoencephalitis world-wide. A major predisposing factor is the profound cellular immune defect caused by HIV infection or other underlying conditions. Cryptococcal meningoencephalitis is fatal unless treated and even with the most advanced treatment it is known for its high mortality rates [1],[2]. Fluconazole (FLC), a triazole antifungal drug, has been the agent most widely used for prophylactic therapy as well as for the long term management of common mycoses such as candidiasis and cryptococcosis owing to its efficacy and safety [3]. Long-term maintenance therapy with azoles creates favorable conditions for the emergence of resistance to the drug and increased azole resistance *in vitro* has been shown to be predictive of treatment failures and infection relapses [4].

The molecular basis of resistance to azole antifungals has been studied extensively in *Saccharomyces cerevisiae* and pathogenic *Candida* species such as *C. albicans* and *C. glabrata* which are phylogenetically distant from *C. neoformans* [5]–[13]. In these fungi, resistance is known to emerge via (1) increased production of multidrug transporters [14]–[16], (2) mutations in ergosterol biosynthetic pathway genes [17],[18], (3) amplification of genomic regions that contain ergosterol biosynthetic pathway genes and transcription factors that positively regulate a subsets of efflux pump genes [19],[20] and (4) activation of Hsp90 that may facilitate the cells to respond to drug stress [21],[22]. In *C. neoformans*, FLC resistant strains have rarely been reported and the emergence of resistance has most often been documented with clinical outcomes of AIDS patients receiving azole maintenance therapy [23]–[27]. The mechanism of resistance in *C. neoformans* during maintenance therapy is poorly understood.

An intriguing pattern of intrinsic azole resistance termed ‘heteroresistance’ was reported in 1999 among *C. neoformans* strains isolated from AIDS patients undergoing FLC maintenance therapy [28] and has only recently been characterized further [29]. This phenomenon of heteroresistance has been described as the emergence of a resistant minor subpopulation, within the single colony of a susceptible strain, that can tolerate concentrations of FLC higher than the strain's MIC. The resistant subpopulations can adapt to increasing concentrations of the drug in a stepwise manner. However, this acquired resistance to high concentrations of FLC is lost during repeated passage in drug free media and the clones return to their original level of heteroresistance. The level of heteroresistance to FLC (LHF) was defined as the lowest concentration of the azole drug at which resistant subpopulations emerge [29]. All strains of *C. neoformans* tested in our laboratory thus far have exhibited different LHF regardless of whether they are pre- or post therapy strains and the frequency of resistant subpopulations that emerge at each LHF ranged between 0.3 and 10% depending on strains [29]. Purification of a homogeneously sensitive subpopulation was not achieved at each strain's LHF while a homogeneous population of resistant cells could readily be obtained by exposure to FLC concentrations equal to or higher than its initial LHF. This acquired resistance to high concentrations of FLC, however, was lost during repeated passage in drug free media and the clones returned to the original LHF at which only 0.3 to 10% of the subpopulations grew. The molecular mechanism involved in this unique pattern of azole resistance remains an enigma.

In this paper, we employed a genomic approach to uncover the mechanism by which *C*. *neoformans* cells acquire resistance to high concentrations of FLC and then subsequently lose the resistance when the drug stress is removed. We demonstrate that the adaptive resistance to higher concentrations of FLC was achieved by duplications of multiple chromosomes in response to drug pressure. Upon repeated transfer in drug free media, cells with multiple disomic chromosomes lose duplicated copies of the chromosomes sequentially and return to their original levels of drug tolerance. Such genomic fluidity that enables the cells to cope with the drug stress was observed in *C. neoformans* strains of both serotypes, A and D. Our results provide an explanation as to the mechanism governing the transiently high azole resistance observed in *C. neoformans*. We propose that this mechanism contributes to the failure of FLC therapy that results in the recurrent infection reported in patients undergoing prolonged azole therapy [28].

## Results

### Characterization of heteroresistance to FLC in strain H99

**Figure 1:**
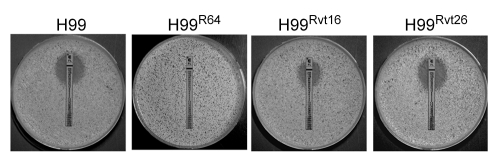
FLC E-tests. Approximately 1×10^6^ cells of each strain were plated on YPD media and E-test strips were placed on the media. The plates were incubated at 30°C for 72 h. Strains: H99 (wild type), H99^R64^ (resistant at 64 µg/ml of FLC), H99^Rvt16^, and H99^Rvt26^ (H99^R64^ derivatives obtained by daily transfer of H99^R64^ on drug-free media for 16 and 26 days, respectively).

All strains of *C. neoformans* tested in our laboratory displayed the intrinsic adaptive heteroresistant phenotype to FLC [29]. Since serotype A strains of *C.neoformans* are the most prevalent of all the four serotypes in clinical settings, we chose the strain H99, a genome sequenced reference strain of serotype A, to study the mechanism of heteroresistance. Equal numbers of colonies were observed on YPD agar media with or without 16 µg/ml FLC. However, growth of the colonies on 16 µg/ml FLC was slightly slower with heterogeneity in size. On YPD media containing 32 µg/ml FLC, only 0.3–0.6% of the input cells consistently formed colonies within 72 h. Therefore, the intrinsic level of H99 FLC heteroresistance was determined to be 32 µg/ml [29]. Exposure of these subclones resistant at 32 µg/ml FLC to stepwise increases in FLC concentration generated clones resistant to 64 µg/ml (strain H99^R64^) and 128 µg/ml (strain H99^R128^). Conversely, repeated transfer in drug free media of cells that had adapted to FLC at concentrations >32 µg/ml resulted in their reversal to original levels of heteroresistance. For instance, the H99^Rvt16^ strain derived from 16 daily transfers of the strain H99^R64^ in drug-free media displayed a FLC resistance phenotype intermediate between H99^R64^ and H99. Its colony size on YPD with 32 µg/ml FLC was larger and its FLC E-test value was higher (48 µg/ml) than the parental H99 strain (E-test MIC = 24 µg/ml, Figure 1). In contrast, the H99^Rvt26^ strain similarly derived from 26 daily transfers of the strain H99^R64^ in drug free media completely reverted back to the parental type.

### Genome analysis of FLC resistant strain H99^R64^

**Figure 2:**
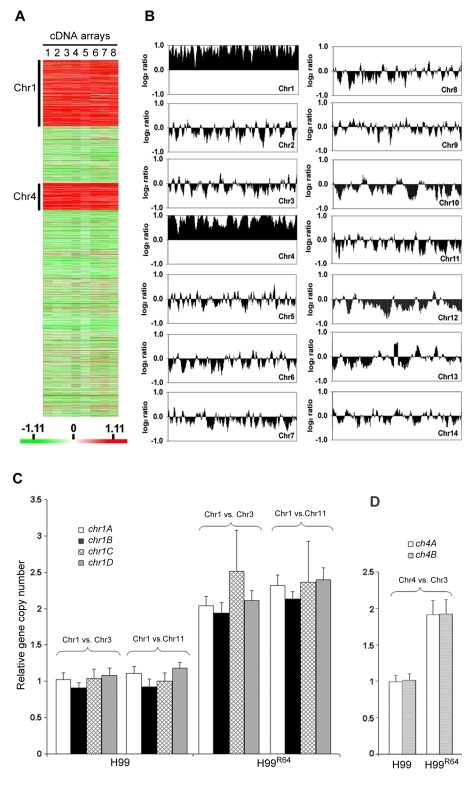
Chromosome 1 and 4 are duplicated in H99^R64^. (**A**) Gene expression profiles. Gene expression patterns of H99^R64^ were compared to those of the wild type strain, H99. A total of eight arrays including three biological repeats and dye-reversed sets were performed as described in materials and methods. Each column (lane 1 to 8) represents a microarray experiment and each row represents the expression of a gene on the array arranged by its chromosomal position. A total of 4149 significant genes were plotted after SAM analysis with a mean FDR of 5%. Chromosomes where upregulated genes are clustered are indicated. The relative expression levels are represented by color as shown in the bar. (**B**) CGH plot of H99^R64^. The genomic DNA of the experimental strain was hybridized against the genomic DNA of the reference strain, H99. Each panel represents the CGH plot of each chromosome. Chromosome number is indicated in the right side corner of each panel. The x-axis represents the position of each gene arranged in the order of its chromosomal location. The y-axis plots gene copy number as a running average over seven genes calculated from the log_2_ ratio of relative hybridization intensity. (**C**) Copy number of four genes on Chr1 determined by qPCR. Four probes (*chr1A*, *chr1B*, *chr1C*, and *chr1D*) at different locations on Chr1 were compared to two control probes; one located on Chr3 (*chr3A*) and the other on Chr11 (*chr11A*) in H99 and H99^R64^, respectively. (**D**) Copy number of Chr4 genes (*chr4A* and *chr4B*) was compared to that of the probe (*chr3A)* on the endogenous control Chr3 in H99 and H99^R64^.

It is possible that the resistant strain H99^R64^ may express a different set of genes compared to H99. Thus, we compared the gene expression profiles of H99^R64^ and H99 using microarray analysis. Of the 6719 detectable genes analyzed, 4149 genes were identified as significant by a mean false discovery rate (FDR) of 5% with significance analysis of microarray (SAM) as described in Material and Methods. We found 763 genes to be up or down regulated at least 1.8-fold in H99^R64^ compared to H99. As expected, some of the differentially regulated genes are annotated for drug-related functions such as ABC transporter, multidrug resistance protein and enzymes involved in the ergosterol biosynthetic pathway. More significantly, among the 491 genes observed to be upregulated in H99^R64^, 308 (63%) are located on chromosome 1 (Chr1) and 143 (29%) on chromosome 4 (Chr4), which in collectively comprises 92% of the upregulated genes in H99^R64^ (Figure 2A). Having a majority of the upregulated genes distinctly clustered in two chromosomes, we suspected some chromosomal anomaly in H99^R64^. To examine global genomic changes in H99^R64^, we performed comparative genome hybridization (CGH). Interestingly, CGH analysis of the H99^R64^ strain revealed that the average log_2_ ratio of hybridization signals for Chr1 and Chr4 was significantly above zero (0.84 and 0.89, respectively) across the entire chromosome as shown in Figure 2B. The simplest explanation for this observation was that Chr1 and Chr4 had duplicated in the cells of H99^R64^. We also analyzed H99 and H99^R64^ by flow cytometry and the data suggested that H99^R64^ is not a diploid strain harboring trisomic Chr1 and Chr4 (Figure S1). Consequently, the observed overexpression of the genes on Chr1 and Chr4 in cDNA microarray was due to the increase in copy number of the genes on the two chromosomes.

To confirm this phenomenon of multiple chromosome duplications revealed by the CGH data, quantitative real time PCR (qPCR) of genomic DNA was performed. Four probes representing the four genes at different locations of chromosome 1 that span the left and the right arm (*chr1A*, *chr1B*, *chr1C*, and *chr1D*) were chosen for qPCR using the same genomic DNA used for CGH analysis. qPCR results of each probe on Chr1 were compared to those of the probe on either Chr3 (*chr3A*) or Chr11 (*chr11A*), which served as unduplicated internal controls. As shown in Figure 2C, the copy number of all tested genes at different locations on Chr1 was close to two fold higher than the genes on Chr3 or Chr11 in H99^R64^ (*P*<0.001), while the relative copy number of those genes was close to 1 in H99. This indicated that H99^R64^ has two copies for each of the four genes on Chr1. Similarly, the qPCR results from probes representing two genes on Chr4 (*chr4A* and *chr4B*) showed the dosage of each gene in H99^R64^ to be two fold of that in H99 (Figure 2D; *P*<0.001). These qPCR results corroborated with the CGH data and suggested that chromosomes 1 and 4 in the strain H99^R64^ were disomic and the disomy of these two chromosomes was associated with resistance at 64 µg/ml FLC.

### The number of disomic chromosomes correlates with the level of FLC resistance

**Figure 3:**
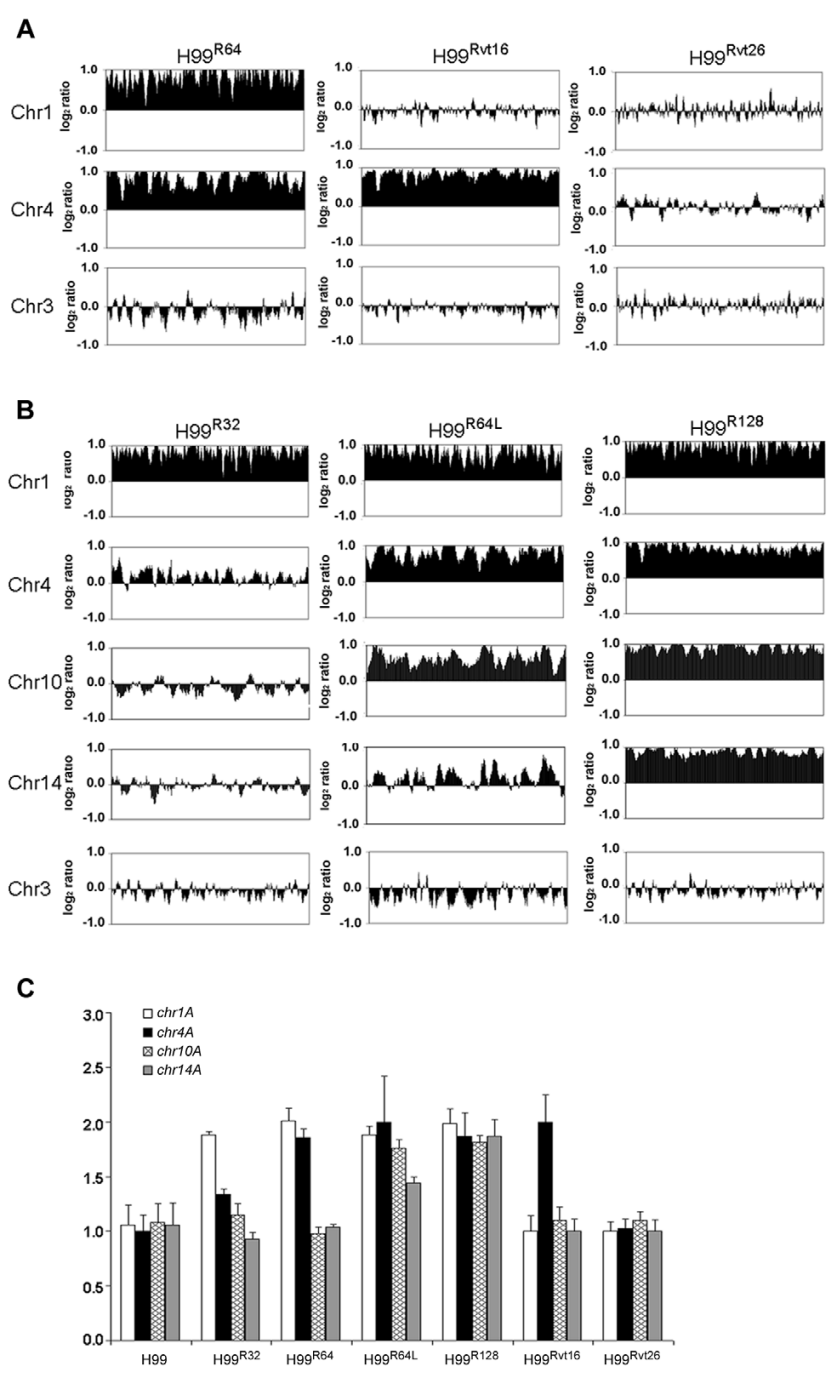
Gain and loss of FLC resistance positively correlates with the number of chromosomes duplicated. (**A**) CGH plots of Chr1, 4, and 3 for strains H99^R64^, H99^Rvt16^, and H99^Rvt26^. (**B**) CGH plots of Chr1, 4, 10, 14, and 3 for strains H99^R32^, H99^64L^, and H99^R128^. (**C**) Copy number of four genes in H99-derived strains with different levels of resistance. The relative gene copy number of four probes (*chr1A*, *chr4A*, *chr10A* and *chr14A*) located on Chr1, 4, 10, and 14 was compared to the control probe (*chr3A*) on Chr3. The same genomic DNA from the strains used for CGH arrays was used for qPCR assays. Only the chromosomes involved in duplication are shown. Chr3 serves as a non-duplicated chromosome control.

Since the resistant clones can adapt to different levels of FLC concentration, it is possible that the FLC resistance level of each clone positively correlates with the number of disomic chromosomes. To test this hypothesis, we analyzed by CGH array six other H99-derived strains that had adapted to different levels of FLC concentration. First, we tested two of the aforementioned reverted strains, H99^Rvt16^ and H99^Rvt26^, which had resulted from repeated transfer of H99^R64^ in drug free media for 16 days and 26 days respectively (Figure 1). CGH data revealed the intermediate revertant H99^Rvt16^ to be monosomic for Chr1 but still disomic for Chr4 while the complete revertant, H99^Rvt26^, contained no disomic chromosomes (Figure 3A and Figure S2). These results suggested that removal of drug pressure caused a loss of the duplicated copies of chromosomes in the cells starting with that of Chr1 and then eventually return to the wild type status. Second, we performed CGH analysis of the H99^R32^ and H99^R128^ strains which were resistant to 32 µg/ml and 128 µg/ml FLC, respectively. CGH plots revealed that only Chr1 was duplicated in H99^R32^, while four chromosomes (Chr1, 4, 10, and 14) were duplicated in H99^R128^ (Figure 3B and Figure S2). Third, we analyzed strain H99^R64L^, a clone of H99^R64^ that was maintained for an additional two weeks on the media with 64 ug/ml FLC.. As was the case with H99^R64^, Chr1 and Chr4 were duplicated in H99^R64L^. Interestingly, Chr10 was also duplicated in H99^R64L^ and the copy number of many genes on Chr14 increased although not quite two-fold compared to that of H99 (Figure 3B). It appears that prolonged incubation of cells at high FLC concentrations results in the emergence of additional disomic chromosomes.

CGH results were confirmed by qPCR analysis of a gene chosen from each of the four chromosomes 1, 4, 10 and 14. As shown in Figure 3C, relative copy numbers of each gene against the internal control gene on Chr3 corroborated the CGH analysis. All four genes located on different chromosomes were duplicated in the strain H99^R128^ while no gene duplication was evident in the complete revertant strain H99^Rvt26^ (*P*<0.001). In the strain H99^R64L^, the gene copy number on Chr10 and Chr14 was close to 2 and 1.5, respectively. Furthermore, chromosome duplication was also verified by quantitative Southern blot analysis using a probe from each of the four affected chromosomes (Figure S3 and Table S1). Collectively, these data strongly suggested that the number of disomic chromosomes positively correlated with the levels of FLC resistance of the strain and with the duration of exposure to FLC.

### Genome fluidity reflected in the gene dosages at colony level

**Figure 4:**
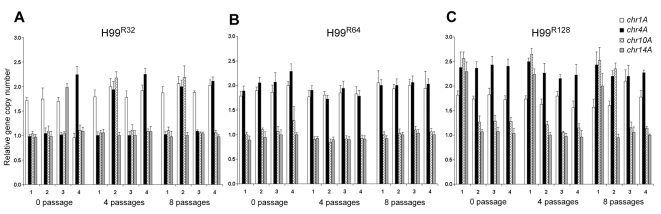
Gene duplication determined by colony qPCR. Gene dosage was determined by qPCR in four independent colonies of H99 derived strains by stepwise selection on YPD agar plates containing increasing concentrations of FLC: (**A**) 32 µg/ml; (**B**) 64 µg/ml; (**C**) 128 µg/ml. The relative gene copy number of four probes (*chr1A*, *chr4A*, *chr10A* and *chr14A*) located on Chr1, 4, 10, and 14 was compared to the control probe on Chr3 (*chr3A*). The 0 passage samples represent initial colonies that appeared 4 days after plating the parental strains on plates containing FLC. The gene duplication in those colonies was also determined following 4 and 8 passages on agar media with the same concentration of FLC.

Since CGH experiments require relatively large amounts of genomic DNA, each strain was allowed to proliferate for many generations on the drug media in order to obtain enough cells. The CGH data, therefore, represents the average status of the whole population grown on the media containing a certain concentration of FLC for many generations. qPCR was performed to examine gene dosages in the small number of individual resistant clones immediately after their emergence on plates containing high concentrations of FLC. This would determine whether gene duplication occurred during the early stages of growth in which resistance was initially observed at the single colony level. We chose 4 different colonies that appeared 4 days after plating naive H99 cells on media with 32 µg/ml FLC. Four independent colonies resistant at 64 and/or 128 µg/ml of the drug (derived from four different 32 and/or four different 64 µg/ml FLC resistant clones, respectively) were also isolated and analyzed. The CGH data suggested that the chromosome duplication occurs primarily in Chr1, 4, 10 and 14 and thus we focused our colony qPCR analysis only on these four chromosomes, although the duplication event might not be limited to these chromosomes. Interestingly, variations in the gene duplication events on different chromosomes was observed among the independent colonies grown on 32 µg/ml and 128 µg/ml FLC, respectively, but not on 64 µg/ml FLC (0 passage in Figure 4A, 4B, and 4C). To test whether prolonged drug-exposure would alter the outcome of gene duplication, the same sets of the four clones from each concentration of FLC were streaked on media with the same FLC concentration for 4 and 8 passages. Single colonies were then subjected to qPCR which exposed the tremendous variability in the duplication of genes representing different chromosomes. For example, one clone from 32 µg/ml FLC plate (clone #2) initially had duplication of a gene on Chr1. After 4 passages, genes on Chr4 and Chr10 were also duplicated in addition to Chr1 and the status of gene duplication in these chromosomes was the same when tested after 8 passages (Figure 4A). Clone #3 from the 32 µg/ml plate, however, appeared to have genes on Chr1 and Chr14 duplicated initially, but the genes on Chr14 did not remain duplicated after longer exposure to the drug. In contrast, clone #4 did not show the gene on Chr1 duplicated until after 4 passages on 32 µg/ml FLC media while gene on Chr4 was duplicated from the beginning and remained duplicated throughout the 8 passages. Generally, a more consistent pattern of gene duplication was observed with independent clones isolated from the plates containing FLC 64 µg/ml compared to those isolated from 32 µg/ml FLC plates (Figure 4B). Fluctuations in the pattern of gene duplication, however, were also obvious among the colonies grown on FLC 128 µg/ml (Figure 4C). These data clearly showed the plasticity of gene duplication patterns at the single colony level, which could not be depicted clearly in CGH data. It is likely that CGH results represent the average status of chromosomes in the whole population and the system is not sensitive enough to allow detection of transient chromosomal duplication events in individual colonies. However, the CGH results of H99^R64L^ apparently revealed the intermediate process of Chr14 duplication in which the gene copy number of Chr14 was 1.5 as verified by qPCR using the same batch of DNA (Figure 3B and 3C). Taken together, our data suggested that when *C. neoformans* was treated with FLC, the process of multiple chromosome duplication may vary among individual cells and the status of chromosome copy number determined by CGH appears to be an average of the whole population from cells grown in the presence of FLC for many generations.

### *ERG11* is important for chromosome 1 duplication under azole stress

**Figure 5:**
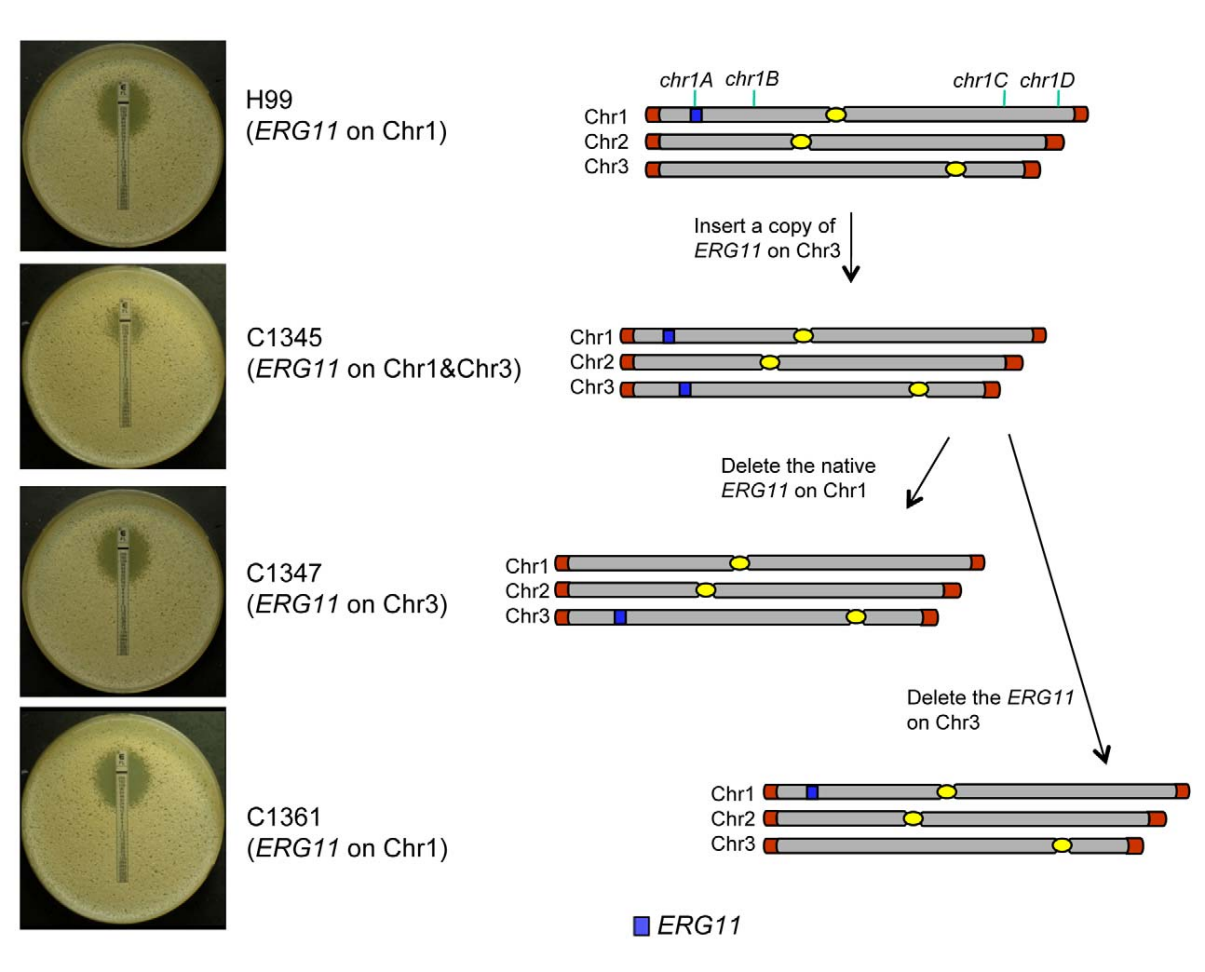
The correlation between *ERG11* location and the phenotype. The diagram on the right represents the chromosomal location of *ERG11* and its corresponding phenotype is shown on the left (FLC E-tests). The position of 4 tested genes (*chr1A*, *chr1B*, *chr1C*, and *chr1D*) on Chr1 is indicated at the top of the diagram.

**Figure 6:**
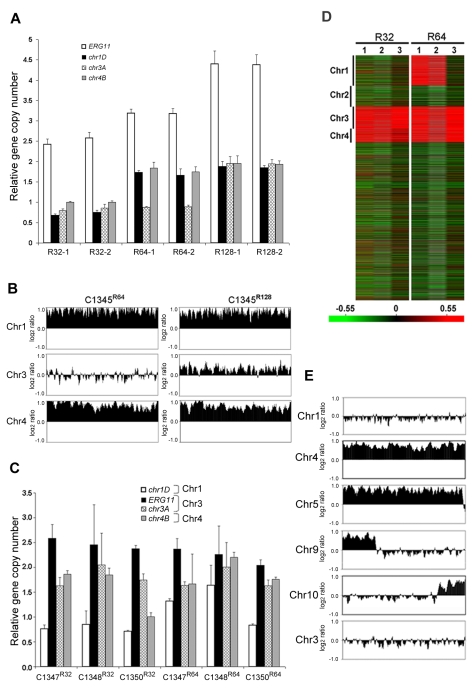
The importance of *ERG11* and *AFR1* in Chr1 duplication. (**A**) Gene duplication patterns in C1345 derived strains. Gene copy number was determined by qPCR in resistant colonies derived from C1345 which contains two copies of *ERG11*, one on Chr1 and the other on Chr3. Two different colonies resistant to FLC 32, 64 and 128 µg/ml are shown respectively. Gene copy number was quantified by measuring four probes (*ERG11* = *chr1A*, *chr1D*, *chr3A* and *chr4B*) located on Chr1, 3 and 4, respectively, in comparison to the control probe (*chr5A*) on Chr5. (**B**) CGH plots of Chr1, 3, and 4 for C1345^R64^ and C1345^R128^ that tolerate 64 and 128 µg/ml FLC, respectively. Only the chromosomes involved in duplication are shown. (**C**) Gene copy number was determined by qPCR in resistant colonies derived from three independent transformants (C1347, C1348, and C1350), each with a single copy of *ERG11* on Chr3. Strains resistant to 32 and 64 µg/ml FLC are shown. The probes located on Chr1, 3, and 4 were compared to the control probe (*chr5A*) on Chr5. (**D**) Heat map of CGH results: lanes 1–3 represent the CGH array of three independent strains, C1347, C1348, and C1350, resistant at 32 and 64 µg/ml FLC, respectively. The relative copy number is represented by color as shown in the bar. Red indicates that the copy number of the genes is close to 2. (**E**) CGH plots of Chr1, 4, 5, 9, 10, and 3 for the C1371 (*afr1Δ* ^R1^) strain resistant at 1 µg/ml of FLC.

It was plausible that formation of disomic chromosomes in association with FLC resistance was due to the presence of certain genes on the duplicated chromosomes which plays crucial role in the survival of cells under the drug stress. Since Chr1 was universally duplicated in the resistant clones, we focused on Chr1 as the first step to determine whether each duplicated chromosome carries genes that confer selective advantage under azole drug stress. Among the annotated genes on Chr1, *AFR1* and *ERG11* were the two candidate genes that had already been characterized involving FLC resistance in *C. neoformans. AFR1* is an ATP binding cassette (ABC) transporter-encoding gene and have shown to play an important role in azole susceptibility [29],[30]. *ERG11* encodes lanosterol-14-α-demethylase, the target of FLC, and increased expression levels of *ERG11* is associated with increased FLC resistance in several fungi [15]–[18]. *ERG11*, the target of FLC, has been proposed to contribute to isochromosome formation in *C. albicans* [20], so we chose it to address its role in Chr1 duplication. If the presence of *ERG11* were the main cause of Chr1 duplication, *ERG11*-containing chromosome would primarily duplicate regardless of the location of the gene. On the other hand, if other genes besides *ERG11* were equally or more important for the survival in the presence of FLC, Chr1 would remain duplicated even if *ERG11* is relocated from Chr1 to other chromosomes. Since *ERG11* is most likely essential, we first inserted an extra copy of *ERG11* on Chr3, which had not duplicated under any level of drug stress and then deleted *ERG11* from its native location on Chr1 (Figure 5). Strain C1345, which contained two copies of *ERG11* – one on Chr1 and the other on Chr3, exhibited elevated resistance to FLC according to E-test (Figure 5) as well as by growth analysis (100% growth at 32 µg/ml and 0.1% growth on 64 µg/ml in contrast to 0.3–0.6% growth at 32 µg/ml and 0 % growth at 64 µg/ml of H99) compared to H99. These data indicate that the extra copy of *ERG11* inserted onto Chr3 conferred increased FLC resistance. Since C1345 had two copies of *ERG11* mimicking the effect of Chr1 duplication regarding *ERG11* copy number, it was of great interest to determine the status of chromosome duplication in C1345 upon exposure to high concentration of FLC. First, qPCR was performed on two independent colonies of C1345 isolated immediately after emerging on the plate containing 32 µg/ml FLC. We detected close to two copies of *ERG11* (*chr1A* probe) but only one copy of other genes on Chr1, Chr3, and Chr4 suggesting no duplication of chromosomes of C1345 at 32 µg/ml FLC (Figure 6A). This is in contrast to H99 subclones resistant to 32 µg/ml FLC in which Chr1 is duplicated (Figure 3B and 3C). However, qPCR of two C1345 colonies isolated directly from 64 µg/ml of FLC plate showed the existence of three copies of *ERG11* (*chr1A* probe) and two copies of both *chr1D* and *chr4A*, but only one copy of *chr3A* (Figure 6A). These data suggested that both Chr1 and Chr4 were duplicated in the C1345 colonies grown in 64 µg/ml of FLC. CGH analysis of the entire cell population harvested from 64 µg/ml FLC (C1345^ R64^) clearly showed duplication of Chr1 and Chr4 and not Chr3 (Figure 6B). In addition, C1345^R128^, the C1345 strain grown on128 µg/ml FLC showed similar chromosome duplication patterns as C1345^R64^ maintaining duplication of Chr1 and Chr4. It is intriguing, however, to observe intermediate hybridization signal for Chr3 in C1345^R128^ (average log_2_ ratio of −0.035 and 0.317 for C1345^ R64^ and C1345^R128^, respectively). qPCR using the same DNA showed that the copy number of *chr3A* probe located on Chr3 was 1.22±0.06, confirming the CGH results. This data suggests that a proportion of the cells in the population of C1345^R128^ strain may have an extra copy of Chr3. Colony PCR from two independent colonies of C1345^R128^ supported duplication of the gene on Chr3 (Figure 6A). The second copy of *ERG11* resides on Chr3 in C1345^R128^ which has not been observed to be duplicated in other strains tested so far. These results showed that two copies of *ERG11,* one on Chr 1 and the other on Chr3 prevented disomy formation of Chr1 at 32 µml FLC but did not prevent disomy formation of Chr1 and Chr4 at FLC 64 µg/ml, a concentration which is 2-fold higher than the level tolerated by C1345. Furthermore, Chr1 was preferentially duplicated over Chr3 at high concentrations of FLC when the *ERG11* existed on both chromosomes.

The *ERG11* gene on the Chr1 was subsequently deleted from C1345 leaving only one copy of *ERG11* inserted on Chr3. The FLC resistance levels of three independent transformants (C1347, C1348, and C1350) were comparable with H99 (100% growth at 16 µg/ml and 0.3–0.6% growth at 32 µg/ml), indicating that translocation of *ERG11* from Chr1 to Chr3 did not alter the strain's FLC resistance level. Clones of these three independent transformants grown in 32 µg/ml FLC were subjected to colony qPCR. Noticeably, the copy number of *ERG11* (*chr1A* probe) and *chr3A* were close to two fold, while the copy number of *chr1D* remained close to one suggesting duplication of Chr3 but not Chr1 (Figure 6C). CGH analysis of C1347^R32^, C1348^R32^, and C1350^R32^ showed Chr3 was duplicated in all three strains (Figure 6D). Interestingly, Chr4 was also duplicated in all three strains although colony PCR results suggested duplication of a gene on Chr4 only in C1347^R32^ and C1348^R32^ (Figure 6C and 6D). This result was different from H99^32R^ in which only Chr1 duplication was observed at 32 µg/ml FLC (Figure 3B). These data suggested that when only one copy of *ERG11* is present in the genome, the *ERG11* bearing chromosome is the primary one to be duplicated at 32 µg/ml FLC. However, additional chromosome duplication (Chr4) was required to tolerate the stress exerted by FLC when *ERG11* was moved from its native location to Chr3. Additional CGH was performed using strains derived from C1347, C1348, and C1350 resistant to 64 µg/ml FLC (Figure 6D). C1347^R64^ and C1348^R64^ displayed disomies of Chr1, Chr3 and Chr4 while C1350^R64^ showed duplication only in Chr3 and Chr4 but not in Chr1. Single colony qPCR of these three strains supported the CGH results although the copy number of *chr1D* in C1347^R64^ was only 1.32 (±0.05), suggesting that Chr1 amplification occurred in a certain portion of the clonal population (Figure 6C). These data indicated that when a single copy of *ERG11* gene exists in the genome, the chromosome carrying *ERG11* is consistently duplicated in all subsequently derived FLC-resistant strains. However, our data also pointed out that *ERG11* was not the sole reason for the Chr1 duplication and duplication of other genes on Chr1 and those on Chr4 also appeared to have contributed to the survival of cells at 64 µg/ml FLC.

### *AFR1* also plays a role in chromosome 1 duplication under FLC stress

In an attempt to investigate other genes on Chr1 that confer resistance to FLC via chromosome duplication, we investigated *AFR1*. Several lines of evidence have indicated that *AFR1* plays an important role in FLC resistance. First, *AFR1* expression was upregulated in both H99^R64^ and the H99 strains treated with FLC. Second, deletion of *AFR1* resulted in a drastic decrease in FLC resistance in H99 [29]. Third, high expression level of *AFR1* resulted in the increased level of FLC resistance [30]. The *afr1Δ* strain (C1371) was used to determine the possible involvement of *AFR1* in Chr1 duplication under FLC stress. If *AFR1* were important for duplication of Chr1, we would not expect Chr1 to be duplicated in *afr1Δ* strains resistant to FLC. The H99 *afr1Δ* strain is extremely sensitive to FLC (MIC 0.38 µg/ml) and its level of heteroresistance was reduced from 32 µg/ml to 1 µg/ml [29]. CGH analysis of the subpopulation resistant at 1 µg/ml FLC (*afr1Δ* ^R1^) clearly showed that Chr1 was not duplicated in the *afr1Δ* ^R1^ strain (Figure 6E). Instead, Chr4 and Chr5 were duplicated along with short segmental duplications of Chr9 and Chr10. Thus, absence of *AFR1* on Chr1 not only abrogated Chr1 duplication but also caused whole duplications or segmental duplication in other chromosomes at 1 µg/ml FLC. Such a chromosomal duplication pattern was presumably due to the presence of genes on these duplicated chromosomes which might compensate for the effect of *AFR1* deletion from Chr1 in *afr1Δ* ^R1^. It is noteworthy that although *ERG11* is present on Chr1 in *afr1Δ* ^R1^, disomy formation of Chr1 does not occur at 1 µg/ml FLC. However, CGH analysis of the subpopulation resistant at 8 µg/ml (*afr1Δ* ^R8^) showed that Chr1 was duplicated along with an additional four chromosomes (Chr 4, 5, 6, and 10; Figure S4). These findings underscore the importance of both *ERG11* and *AFR1* in the formation of Chr1 disomy under FLC stress.

### Disomic chromosome formation is a common phenomenon in strains of *C. neoformans*

All strains of *C. neoformans* tested thus far displayed the FLC heteroresistant phenotype [29]. Although different strains displayed heteroresistance at different concentrations of FLC, the stepwise exposure to higher concentrations of FLC allowed the strains to adapt to levels of FLC that are higher than their original MIC. These resistant strains all reverted to the original level of resistance upon removal of drug pressure. To investigate whether chromosome duplication associated with FLC resistance was an H99-specific event, we analyzed a number of matched pairs of naive vs. FLC-adapted resistant isolates in both serotype A and D backgrounds. Consistent with the observation in H99, FLC-resistant strains derived from both serotype backgrounds contained disomic chromosomes according to CGH analysis (Figure S5), even though the duplicated chromosomes were not always identical in these strains. These results demonstrated that chromosome duplication associated with FLC resistance is a general mechanism employed by *C. neoformans* to overcome the stress exerted by FLC.

## Discussion

We report here that *C. neoformans* consistently forms disomies in multiple chromosomes in response to high level of azole pressure in both serotype A and D strains. Duplicated copies of the disomic chromosomes are lost as the drug pressure is removed. While there can be minor variations in the number of duplicated chromosomes among individual colonies grown on the same FLC media, the number of disomic chromosomes in the population of the overall cultures positively correlates with the adaptation to stepwise increase in FLC concentration.

Aneuploidy associated with azole resistance was reported in *Candida albicans* where a substantially higher frequency of aneuploidy was found among azole resistant strains compared to susceptible strains [19]. In addition, chromosome instability, specific segmental aneuploidy, translocation of chromosomal arms and whole chromosome duplication have been previously reported in *Candida* species [20],[31],[32].

One could argue that the clones with disomy observed in the subpopulation of H99 under FLC stress may comprise a normal population that is selected by the drug rather than the drug induced chromosome amplification. There are three reasons for this argument being unlikely. First, aneuploidy caused by chromosome missegregation occurs once every 5×10^5^ cell divisions in yeast [33] and once every 10^4^ to 10^5^ cell divisions in mammalian cells [34]. The frequency of FLC resistant clones of H99 (0.3 to 0.6%) that emerged on drug containing media is too high to be the result of spontaneous chromosomal missegregation. Furthermore, the frequency of FLC resistant clones in different strains can be as high as 10% [29]. The frequency at which aneuploidy occurs in *C. neoformans* under FLC stress, therefore, is several logs higher than the frequency of spontaneous aneuploidy formation in other eukaryotes. Second, H99 is the most widely studied strain of *C. neoformans* and yet a clone derived from H99 that contains disomic chromosomes in a stress-free environment has never been reported. Third, we observed disomy formation in H99 only when exposed to FLC but not other xenobiotics such as trichostatin A, gliotoxin or rhizoxin (data not shown). Aneuploidy is reported to have multiple effects on cellular physiology and cell division in haploid yeast [35]. Consistent with findings in yeast, disomic chromosomes in *C. neoformans* result in a proliferative disadvantage as evidenced by the retarded growth rate of H99^R64^ which harbors extra copies of Chr1 and 4, and exhibits lower virulence in mice compared to the wild type strain (Figure S6). Although many fungi undergo chromosome length polymorphisms, chromosomal loss [36] or gain of minichromosomes [37] under different environmental stress, the degree of consistency and reproducibility of genomic fluidity observed in the present work has not been reported in other fungi.

Since genetically identical cells of a single *C. neoformans* colony exposed to a high concentration of FLC can produce small subpopulations that show a marked difference in FLC susceptibility, we can speculate that this variability is linked to stochasticity in gene expression [38]. The genes that govern the capacity to differentiate into heteroresistant subtypes are not known. Although the CGH data show an increase of specific disomic chromosomes when *C. neoformans* is challenged by increasing drug pressure, minor variations in duplicated chromosomes appear to occur among individual colonies. Such plastic outcomes of duplication events can be advantageous for *C. neoformans* since it can provide the flexibility required for the cells to respond to various kinds of sudden stress it encounters either in the environment or in the host. The extra copy of a disomic chromosome may have resulted from non-disjunction, which occurs commonly in eukaryotes under different stresses [39],[40]. In mammalian systems, inhibition of cholesterol biosynthesis by blocking sterol 14 α-demethylase (*ERG11* ortholog) induces the formation of polyploid cells and mitotic aberrations [41]. Since ergosterol, the counterpart of cholesterol in fungi, is the essential molecule for maintaining membrane integrity, depletion of ergosterol in nuclear and cell membranes due to FLC treatment may jeopardize normal patterns of cytokinesis and enhance the frequency of chromosomal non-disjunction. For example, the spindle pole body (SPB), a fungal equivalent of the centrosome is closely associated with the outer nuclear membrane in *C. neoformans* [42]. Once integrity of the nuclear membrane is compromised by depletion of ergosterol in FLC treated cells, segregation of the SPB may become irregular and enhance the chromosomal instability during cell division [43].

Gene duplication is known to be one of the key mechanisms which allows fungi to be selected during evolution [44]. Aneuploidy resulting in gene duplication has been reported to be the initial evolutionary change in *S. cerevisiae* selected in vitro to overcome loss of the myosin II protein which is crucial for normal cytokinesis [45].

In response to drug pressure, disomic chromosomes that contain genes relevant to ergosterol synthesis and drug transport could be beneficial for the survival of *C. neoformans.* Our hypothesis on the crucial roles of *ERG11* and *AFR1* in the occurrence of Chr1 duplication in clones resistant to high drug concentrations was borne out. When grown on 32 ug/ml FLC, the drug level at which Chr1 disomy occurs in H99, the strain with *ERG11* translocated from Chr1 to Chr3 showed duplication only in Chr3 but not in Chr1. However, an extra copy of *ERG11* on Chr3 in addition to the native copy on Chr1 was not enough to prevent Chr1 duplication at FLC concentrations higher than 32 µg/ml. This indicated that multiple copies of *ERG11* alone can not meet the challenge of very high FLC stress. Similarly, Chr1 was not duplicated when *AFR1* was deleted and grown on 1 µg/ml FLC (the strain's initial heteroresistance level). However, Chr4 and Chr5 were duplicated along with short segmental duplications of Chr9 and Chr10, which most likely compensate for the loss of *AFR1*. These findings underscore the important roles of *ERG11* and *AFR1* in Chr1 duplication under drug stress. Afr1 is related to Snq2 of *C. glabrata* which is known to function as a transporter for several compounds including FLC [46]. In our test, *afr1Δ* was also sensitive to cycloheximide and rhizoxin treatment suggesting that *AFR1* may function as a transporter for these drugs (data not shown). An ideal experiment to test the hypothesis that duplication of Chr1 causes drug resistance would be to construct a strain in which only Chr1 is duplicated without exposure to azoles and then test the FLC resistance level of the strain. In *S. cerevisiae*, strains containing duplicated chromosomes could be constructed and the effect of aneuploidy tested [35]. Currently, construction of such strains, however, is technically not feasible in *C. neoformans*. Duplication of Chr1 has never been observed in H99 prior to the acquisition of FLC resistance. Since the resistance persisted as long as Chr1 disomy remained but was lost simultaneously after prolonged maintenance in drug free media, we are convinced that the two genes contribute to disomy of Chr1.

The *C. neoformans* genome contains all the genes known to be associated with ergosterol biosynthesis and has twice as many drug-related transporters as *S. cerevisiae*. These genes are distributed widely among 14 chromosomes and it is possible that some of them play a role in azole tolerance. It remains to be determined whether any other gene and its regulator necessitate duplication of the chromosome on which it resides. *C. neoformans* strains, regardless of the chronology of isolation either before or after the launch of azole drugs, showed that 0.3 to 10% of the subpopulations consistently resisted FLC concentrations higher than their MICs [29]. This number did not vary significantly during repeated tests. Although FLC resistant strains of *C. neoformans* have been increasingly reported from azole therapy failure cases [24], [26], [28], [29], [47]–[49], the number of stable FLC resistant mutants among clinical isolates is rare compared to other pathogenic fungi [15],[50]. One reason for the rarity in isolating FLC resistant *C. neoformans* mutants may be that heteroresistance masks mutation. The regular mutation rate is 10^−5^ to 10^−6^ and such a low population would be masked by the adaptive heteroresistant population. Our results provide the foundation for a mechanistic understanding of transient high azole resistance to FLC which might occur during prolonged maintenance therapy with azoles.

## Materials and Methods

### Strains and media

*C. neoformans* isolates H99 and NIH376 are serotype A strains; NIH429 is serotype D [29]. Table 1 lists all the H99 derived strains used in this study. Strains were stored in 25% glycerol stocks at −80°C until use and were maintained on YPD (1% yeast extract, 2% peptone, 2% glucose) agar plates at 30°C for routine cultures.

**Table 1:** Strains used in the study.

Strain name	Descriptions
H99	wild type; resistant to 16 µg/ml FLC
H99^R32^	derived from H99; resistant to 32 µg/ml FLC
H99^R64^	derived from H99^R32^; resistant to 64 µg/ml FLC
H99^R64L^	H99^R64^ maintained on 64 µg/ml FLC for long period of time
H99^Rvt16^	H99^R64^ transferred 16 times in drug free media; resistant to 16 µg/ml FLC (see Materials and Methods)
H99^Rvt26^	H99^R64^ transferred 26 times in drug free media; resistant to 16 µg/ml FLC (see Materials and Methods)
H99^R128^	derived from H99^R64^; resistant to 128 µg/ml FLC
C1345	two copies of *ERG11*; one on Chr1 and one on Chr3
C1345^R64^	derived from C1345; resistant to 64 µg/ml FLC
C1345^R128^	derived from C1345^R64^; resistant to 128 µg/ml FLC
C1347	derived from C1345 with *ERG11* deletion on Chr1
C1348	derived from C1345 with *ERG11* deletion on Chr1
C1350	derived from C1345 with *ERG11* deletion on Chr1
C1347^R32^	derived from C1347; resistant to 32 µg/ml FLC
C1348^R32^	derived from C1348; resistant to 32 µg/ml FLC
C1350^R32^	derived from C1350; resistant to 32 µg/ml FLC
C1347^R64^	derived from C1347^R32^; resistant to 64 µg/ml FLC
C1348^R64^	derived from C1348^R32^; resistant to 64 µg/ml FLC
C1350^R64^	derived from C1350^R32^; resistant to 64 µg/ml FLC
C1371	derived from H99 with *AFR1* deletion
*afr1Δ* ^R1^	derived from C1371; resistant to 1 µg/ml FLC
*afr1Δ* ^R8^	derived from C1371; resistant to 8 µg/ml FLC
NIH376	a serotype A environmental isolate from the NIH collection
NIH429	a serotype D environmental isolate from the NIH collection

### Heteroresistant phenotype

Fluconazole (FLC) was provided as powder by Pfizer Global Research & Development (Groton, CT). Stock solutions were prepared in dimethyl sulfoxide (Sigma) at a concentration of 50 mg/ml. Analysis of FLC heteroresistance was performed by the method described previously [29]. Briefly, cell suspensions (1×10^3^ to 4×10^3^ CFU/ml) in sterile saline were plated on YPD plates containing various concentrations of FLC. Growth was recorded after 72 h incubation at 30°C. Isolates were considered to be heteroresistant when resistant clonal populations were able to grow on a plate containing FLC. Resistant subpopulations were exposed to stepwise increases in FLC concentrations on YPD media.

### Gene expression array analysis

Microarray slides were purchased from the Genome Sequencing Center at Washington University, St Louis. For cDNA arrays, overnight cultures were diluted to OD_600_ ≅ 0.2 and grown in YPD liquid media for 7 hr. RNA was extracted from yeast cells using Trizol (Invitrogen, Carlsbad, CA), and purified with RNeasy MinElute cleanup kit (Qiagen, Valencia, CA). RNA was labeled and hybridized as described previously [51]. Arrays were scanned on a GenePix 4000B scanner and analyzed using GENEPIX PRO 6.0 (Axon Instruments, Foster City, CA). Data were further analyzed in mAdb database at http://madb.niaid.nih.gov. Three biological repeats were performed using three independent RNA sets isolated from cells cultured on different days and the dye-reverse hybridizations were performed for all 3 sets. One set of RNA was also subjected to technical repeats. All statistically significant genes were identified by significance analysis of microarray using a mean false discovery rate of less than 5%. Only statistically significant genes were used for data analysis. Although the microarray slides used in this study were printed with 70-mers that are designed to uniquely represent each gene in *C. neoformans* serotype D, the oligomers were also optimized for homology to genes predicted in the serotype A strain, H99 (http://genome.wustl.edu/services/microarray/cryptococcus_neoformans).

### Comparative genome hybridization

Genomic DNA was prepared from *C. neoformans* strains grown overnight in 10 ml YPD medium as described previously [52]. 5 µg DNA was digested with *Dpn*II (10 U/µg DNA, New England Biolabs, Ipswich, USA) and labeled with dye according to the BioPrime®Array CGH Genomic Labeling System protocol (Invitrogen Life Technologies, Carlsbad, USA). In all CGH experiments, Alexa647 was used to label DNA from the experimental strains and Alexa555 was used to label DNA from the reference control strain (Invitrogen Life Technologies, Carlsbad, USA). Labeled DNA was purified with the purification kit from the same manufacturer and subjected to competitive hybridization with the 70mers microarray. Sample hybridization and data collection were carried out as described above. Data were further analyzed in mAdb database after applying 50th percentile (Median) normalization.

Two parameters were considered for the CGH experiments. First, we hybridized the slides using H99 genomic DNA as both the experimental and the reference control samples. The scatter plot of the normalized log_10_ signal intensity of both channels showed tight correlation between two probes attesting to the reliability of the hybridization patterns of H99 genomic DNA to the JEC21-based 70mer slides. Second, we tested the reproducibility between arrays. Data from five independent CGH arrays were obtained from the H99 control set (H99-Alexa 555 vs. H99-Alexa 647) as well as from the H99^R64^ set (H99-Alexa 555 vs. H99^R64^-Alexa 647). The data were highly consistent indicating high reproducibility between the arrays. Therefore, in most CGH studies, only one or two arrays per strain were analyzed.

To visualize the CGH array in a chromosomal context, data were imported into Excel format from the mAdb database. CGH data was further normalized by subtracting the average log_2_ signal ratio of each gene obtained in control experiments (H99 Alexa647 vs. H99 Alexa555) from that of a corresponding gene in the experimental data set to compensate for the dye and background bias. Relative hybridization levels were plotted as a running average over seven ORFs and clipped to the range corresponding to 1–2 copies (log_2_ ratio of 0–1, respectively). Each ORF was sorted according to their gene number corresponding to its order along each chromosome (plotted on the x-axis). Although the genomes are largely co-linear between the current genomic assemblies of H99 and JEC21, there are several apparent inversions and translocations. Due to these alterations, homologous chromosomes between the H99 and JEC21 assemblies have been assigned different numbers for some chromosomes [53]. The chromosomal number assignment of H99 was adopted in our CGH data. Due to the translocation events in Chr3, Chr4 and Chr11 of H99, the order of genes on these chromosomes was manually arranged according to its JEC21 counterparts.

### Quantitative real time PCR

To quantify the gene copy number on specific chromosomes in wild-type and FLC-resistant strains, quantitative real time PCR (qPCR) assays were performed. For confirmation of CGH data, the same genomic DNA from strains used in CGH arrays was used for qPCR assays. For individual colony qPCR, genomic DNA of selected colonies was used. For colony DNA extraction, a single colony was picked with a sterile toothpick, suspended in 40 µl of 10 mM EDTA buffer in a microcentrifuge tube, boiled for 6 min and centrifuged. The supernatant was diluted 1∶10 in TE buffer and 5 µl of diluted DNA template was added to 20 µl of the qPCR mix (Applied Biosystems, Branchburg, NJ). The reaction was performed in an Applied Biosystems 7500 Real-Time PCR System. Each reaction was run in triplicate and the average Ct value was converted to relative amount of DNA using the relative standard curve method. The sequences of the primers and probes used for the qPCR are listed in Table S2. The genes *CNAG_02959* on Chr3, *CNAG_00869* on Chr5 and /or *CNAG_07554* on Chr11 were chosen as endogenous controls. For each specific gene, its copy number was obtained by comparing its qPCR value with the endogenous control and expressed as relative gene copy number.

### Gene manipulation

*ERG11* was cloned by PCR and sequenced. The *NAT* selectable marker was cloned into the 5′ flanking region of *ERG11* and the resulting construct was inserted in the intergenic region between *CNAG_03012* and *CNAG_03013* on Chr3 which were generated by PCR and sequenced. The final construct was transformed into H99 and the transformant containing a second copy of *ERG11* between the intergenic region of CNAG_03012 and CNAG_03013 on Chr3 was screened by PCR and confirmed by Southern blot analysis. Subsequently, the *ERG11* gene on Chr1 was deleted with the *NEO* gene from the clone containing two copies of *ERG11* (C1345) by biolistic transformation.

### Statistics

An unpaired *t* test was used for the statistical analysis of qPCR data. A *P* value of less than 0.05 was considered to be significant.

## Supporting Information

Figure S1 FACS analysis and morphology of H99, H99^R32^ and H99^R64^ strains. (A) FACS analysis. Log phase cells of H99, H99^R32^, and H99^R64^ were fixed and subjected to FACS analysis as described (Lengeler, KB, Cox, GM and Heitman, J 2001. Infect and Immun. 69:115-122). Blue, H99; red, H99^R32^; green, H99^R64^. (B) Morphology of H99, H99^R32^, and H99^R64^. The cell size of H99^R32^ and H99^R64^ was larger than H99. In addition, elongated cells were frequently observed in H99^R32^ and H99^R64^. These differences might affect the outcome of FACS and caused the peaks of H99^R32^ and H99^R64^ shifted to the right more than expected.
(0.35 MB TIF)

Figure S2 CGH plots of 14 chromosomes for strains H99^R32^, H99^R64L^, H99^R128^, H99^Rvt16^, and H99^Rvt26^. The genomic DNA of the experimental strain was hybridized against the genomic DNA of reference strain, H99. Each panel represents CGH plot of each chromosome. Chromosome number is indicated in the right side corner of each panel. X-axis represents the position of each gene arranged in the order of its chromosomal location. Y-axis plots gene copy number as a running average over seven genes calculated from log_2_ ratio of relative hybridization intensity.
(1.45 MB XLS)

Figure S3 Quantitative Southern blot analysis. (A) Genomic DNAs were digested with the restriction enzyme *Bgl*I. After fractionation on a 0.8% agarose gel the DNA was transferred onto a Hybond-N nylon membrane (Amersham Biosciences, Buckinghamshire, UK). The membrane was hybridized at 65°C with [α-^32^P] dCTP labeled probes using StripEZ DNA kit (Ambion Inc, Austin, TX). PCR was used to generate probes with the primer pairs listed in Table S3. (B) After hybridization, the membrane was exposed to a phospho-imager screen and signals were quantified with ImageQuant (Molecular Dynamics). The relative copy number of each gene was obtained by comparing the signal intensity of each gene to that of the internal control probe, *chr3*.
(0.31 MB TIF)

Figure S4 CGH plots of *afr1Δ* ^R8^ strain. The *afr1Δ* ^R8^, resistant to 8 µg/ml FLC, was obtained by exposing C1371 (*afr1Δ*) to increasing concentrations of FLC stepwise. The genomic DNA of *afr1Δ* ^R8^ was hybridized against H99 genomic DNA. Data was normalized by subtracting average log_2_ signal ratio of each gene obtained in control experiment (H99-Alexa647 vs. H99-Alexa555) from that of corresponding gene in experimental data set to compensate for the dye and background bias. Each panel represents CGH plot of each chromosome from *afr1Δ* ^R8^ strain. Chromosome number is indicated in the right side corner of each panel.
(0.13 MB TIF)

Figure S5 CGH plot of FLC-resistant strains generated from serotype A and D strains. NIH376 (serotype A) and NIH429 (serotype D) are environmental isolates and genetically unrelated to H99 and JEC21. NIH376^R64^ and NIH429^R64^ are FLC resistant strains derived from NIH376 and NIH429, respectively. The genomic DNA of NIH376R64 was hybridized against the NIH376 genomic DNA using the JEC21-based 70mer slides. Data was normalized by subtracting average log_2_ signal ratio of each gene obtained in control experiment (NIH376-Alexa647 vs. NIH376-Alexa555) from that of the corresponding gene in experimental data set to compensate for the dye and background bias. Same data normalization procedure was applied to NIH429^R64^ using NIH429 as the control experiment. Each panel represents CGH plot of each chromosome from NIH376^R64^ (A), and NIH429^R64^ (B). Chromosome number is indicated on the side of each panel.
(0.35 MB TIF)

Figure S6 H99^R64^ growth rate is slower and its virulence is lower compare to wild type. (A) *In vitro* growth kinetics of H99 and H99^R64^. An overnight culture of each strain was inoculated in duplicate into 50 ml YPD broth at a starting OD_600_ of 0.2. The cells were incubated with shaking at 37°C for 32 h. The OD_600_ of the cultures was measured at various times after inoculation (0, 2, 4, 6, 8, 10, 12, 24, and 32 h). (B) Virulence study of H99 and H99^R64^. The animal study was approved by NIH institutional animal care and use committee. To compare the virulence between H99 and H99^R64^, a murine model of pulmonary cryptococcosis was established in female BALB/c mice (weight, 20 g). Mice were anesthetized with isoflurane and a 20 µl droplet containing 5×10^7^ cells was inoculated by intra-nasal inhalation. Ten animals were used for each strain. The survival of mice was recorded daily for a total of 45 days.
(0.11 MB TIF)

Table S1 Oligonucleotides used for Southern analysis
(0.03 MB DOC)

Table S2 Oligonucleotides used for qPCR assays
(0.04 MB DOC)
