# Supplementary figures and images for: Cryptococcus neoformans Overcomes Stress of Azole Drugs by Formation of Disomy in Specific Multiple Chromosomes
Source: PLoS Pathog. 2010 Apr 1;6(4):e1000848. doi: 10.1371/journal.ppat.1000848 (PMC2848560; doi:10.1371/journal.ppat.1000848)

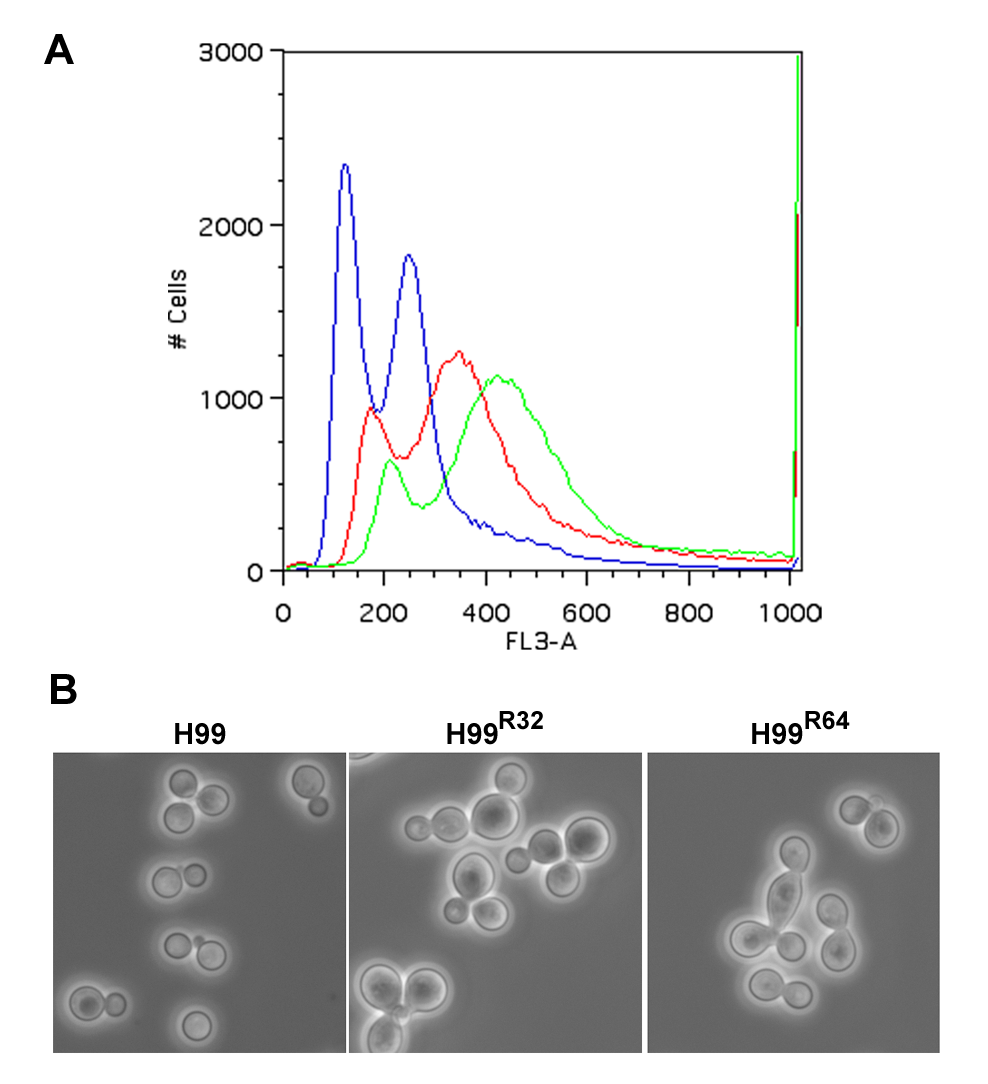

Supplement: Figure S1 — FACS analysis and morphology of H99, H99R32 and H99R64 strains. (A) FACS analysis. Log phase cells of H99, H99R32, and H99R64 were fixed and subjected to FACS analysis as described (Lengeler, KB, Cox, GM and Heitman, J 2001. Infect and Immun. 69:115-122). Blue, H99; red, H99R32; green, H99R64. (B) Morphology of H99, H99R32, and H99R64. The cell size of H99R32 and H99R64 was larger than H99. In addition, elongated cells were frequently observed in H99R32 and H99R64. These differences might affect the outcome of FACS and caused the peaks of H99R32 and H99R64 shifted to the right more than expected. (0.35 MB TIF) [file ppat.1000848.s001.tif]

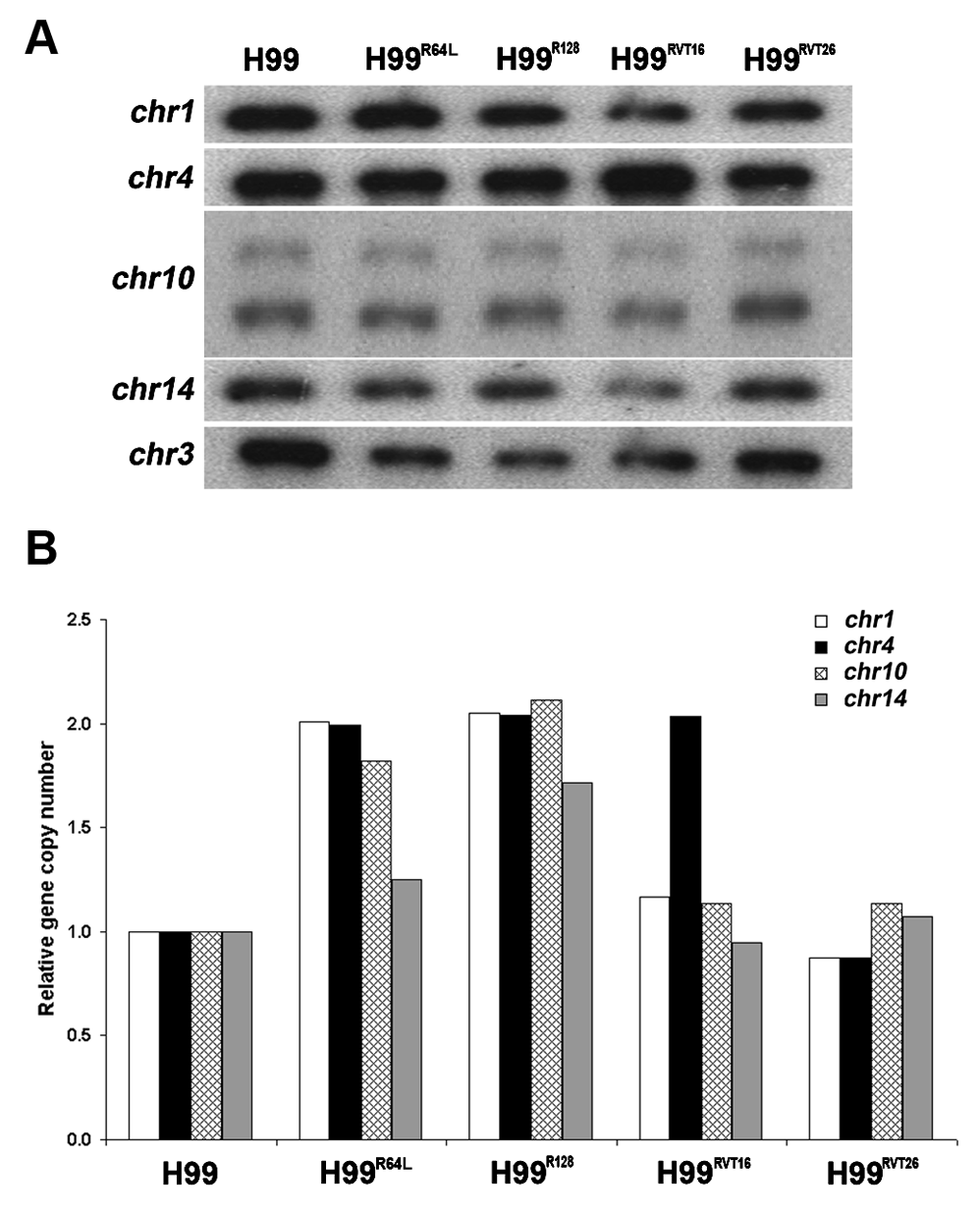

Supplement: Figure S3 — Quantitative Southern blot analysis. (A) Genomic DNAs were digested with the restriction enzyme BglI. After fractionation on a 0.8% agarose gel the DNA was transferred onto a Hybond-N nylon membrane (Amersham Biosciences, Buckinghamshire, UK). The membrane was hybridized at 65°C with [α-32P] dCTP labeled probes using StripEZ DNA kit (Ambion Inc, Austin, TX). PCR was used to generate probes with the primer pairs listed in Table S3. (B) After hybridization, the membrane was exposed to a phospho-imager screen and signals were quantified with ImageQuant (Molecular Dynamics). The relative copy number of each gene was obtained by comparing the signal intensity of each gene to that of the internal control probe, chr3. (0.31 MB TIF) [file ppat.1000848.s003.tif]

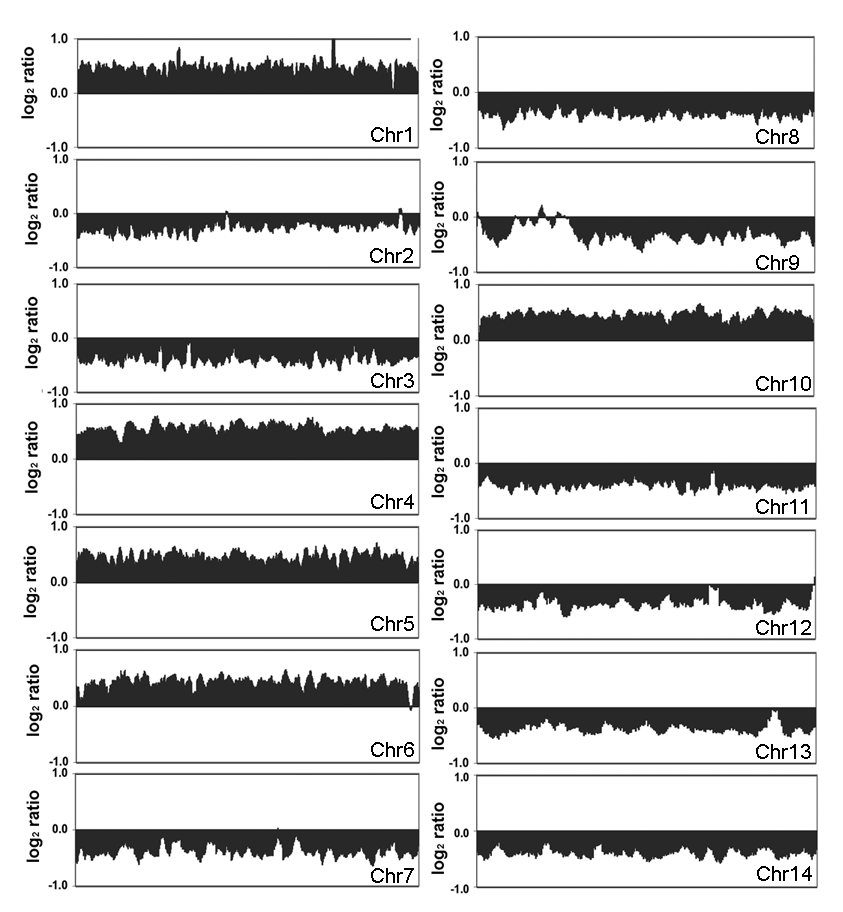

Supplement: Figure S4 — CGH plots of afr1Δ R8 strain. The afr1Δ R8, resistant to 8 µg/ml FLC, was obtained by exposing C1371 (afr1Δ) to increasing concentrations of FLC stepwise. The genomic DNA of afr1Δ R8 was hybridized against H99 genomic DNA. Data was normalized by subtracting average log2 signal ratio of each gene obtained in control experiment (H99-Alexa647 vs. H99-Alexa555) from that of corresponding gene in experimental data set to compensate for the dye and background bias. Each panel represents CGH plot of each chromosome from afr1Δ R8 strain. Chromosome number is indicated in the right side corner of each panel. (0.13 MB TIF) [file ppat.1000848.s004.tif]

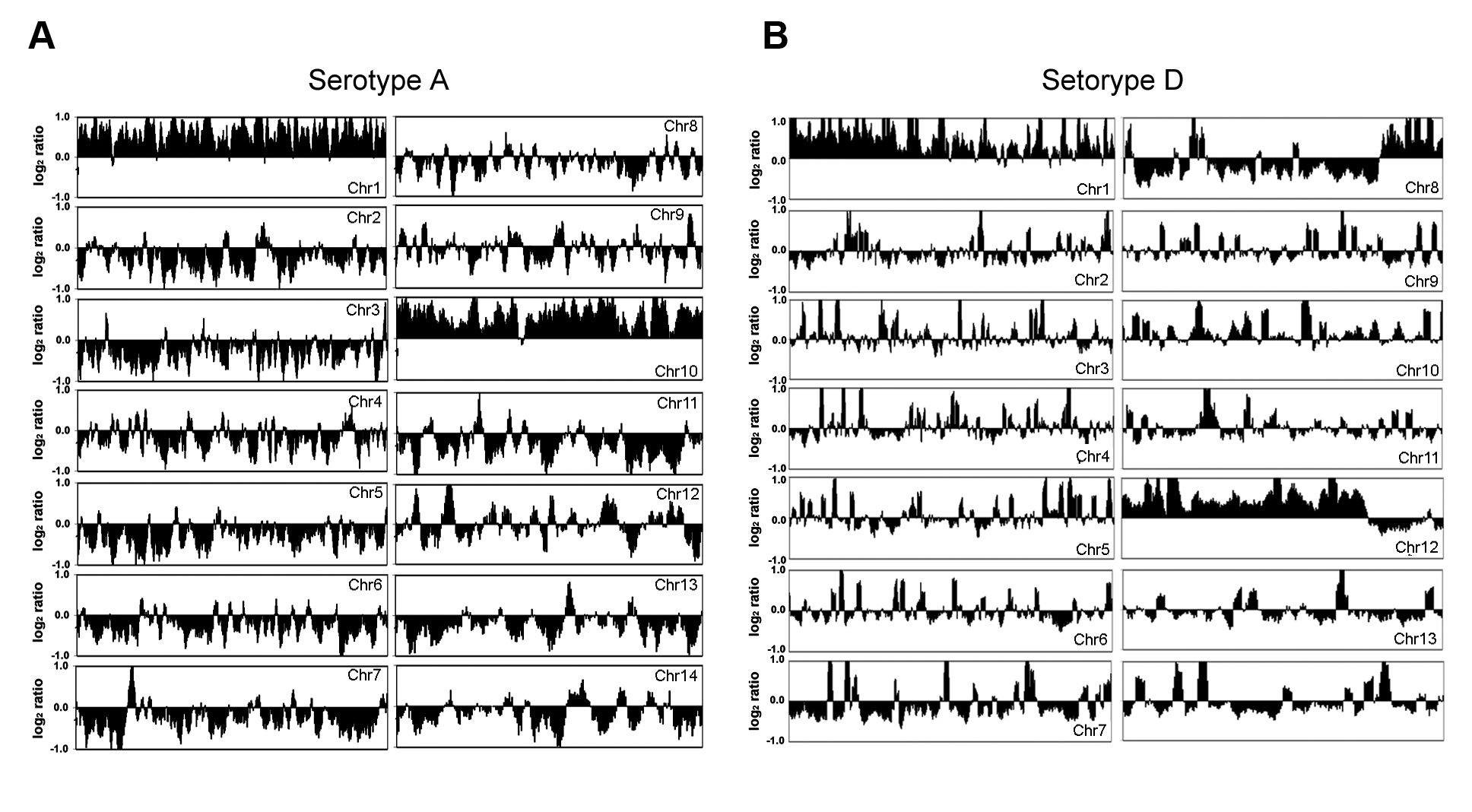

Supplement: Figure S5 — CGH plot of FLC-resistant strains generated from serotype A and D strains. NIH376 (serotype A) and NIH429 (serotype D) are environmental isolates and genetically unrelated to H99 and JEC21. NIH376R64 and NIH429R64 are FLC resistant strains derived from NIH376 and NIH429, respectively. The genomic DNA of NIH376R64 was hybridized against the NIH376 genomic DNA using the JEC21-based 70mer slides. Data was normalized by subtracting average log2 signal ratio of each gene obtained in control experiment (NIH376-Alexa647 vs. NIH376-Alexa555) from that of the corresponding gene in experimental data set to compensate for the dye and background bias. Same data normalization procedure was applied to NIH429R64 using NIH429 as the control experiment. Each panel represents CGH plot of each chromosome from NIH376R64 (A), and NIH429R64 (B). Chromosome number is indicated on the side of each panel. (0.35 MB TIF) [file ppat.1000848.s005.tif]

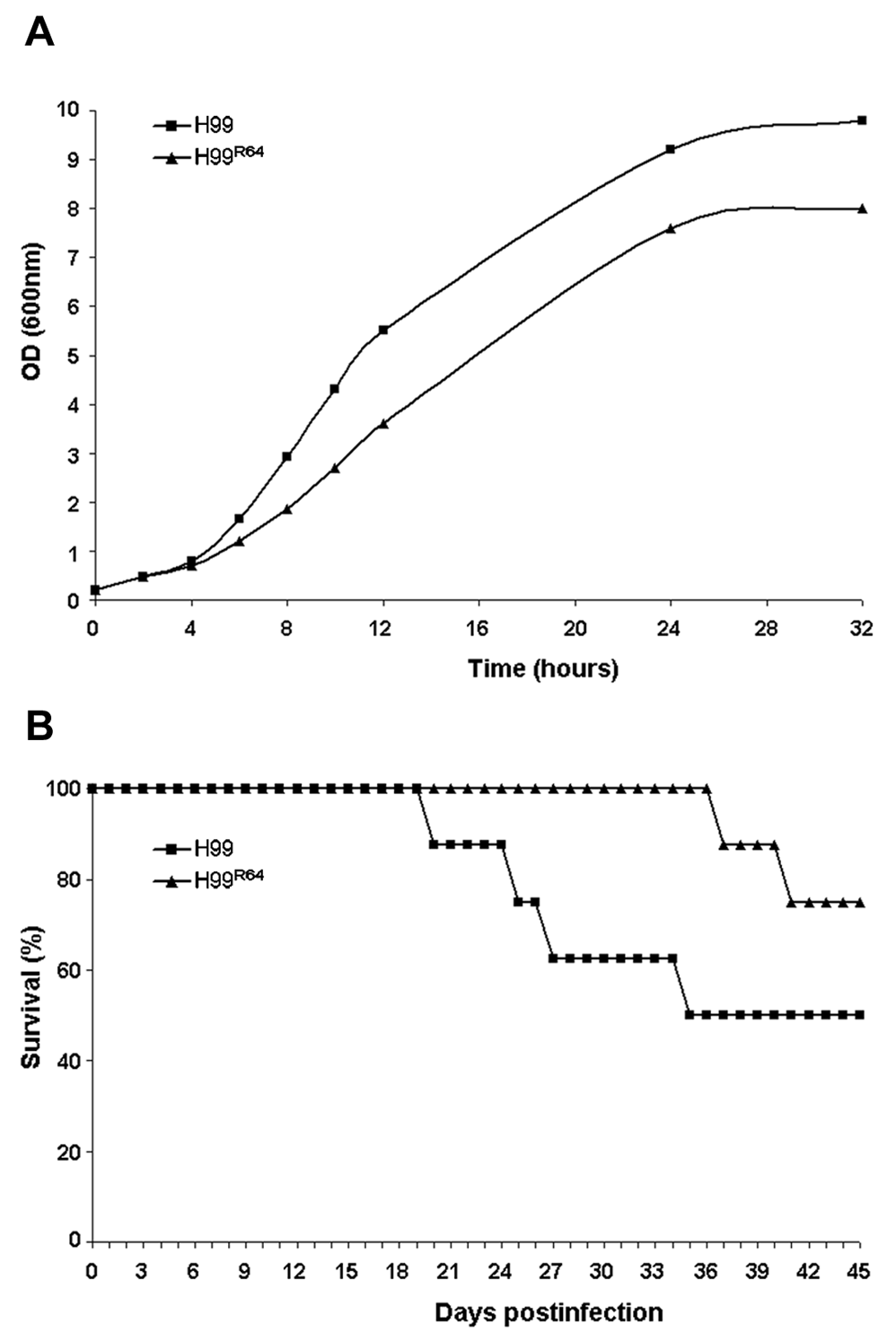

Supplement: Figure S6 — H99R64 growth rate is slower and its virulence is lower compare to wild type. (A) In vitro growth kinetics of H99 and H99R64. An overnight culture of each strain was inoculated in duplicate into 50 ml YPD broth at a starting OD600 of 0.2. The cells were incubated with shaking at 37°C for 32 h. The OD600 of the cultures was measured at various times after inoculation (0, 2, 4, 6, 8, 10, 12, 24, and 32 h). (B) Virulence study of H99 and H99R64. The animal study was approved by NIH institutional animal care and use committee. To compare the virulence between H99 and H99R64, a murine model of pulmonary cryptococcosis was established in female BALB/c mice (weight, 20 g). Mice were anesthetized with isoflurane and a 20 µl droplet containing 5×10^7^ cells was inoculated by intra-nasal inhalation. Ten animals were used for each strain. The survival of mice was recorded daily for a total of 45 days. (0.11 MB TIF) [file ppat.1000848.s006.tif]
