# Supplementary material for: Cryptococcus neoformans Overcomes Stress of Azole Drugs by Formation of Disomy in Specific Multiple Chromosomes
Source: PLoS Pathog. 2010 Apr 1;6(4):e1000848. doi: 10.1371/journal.ppat.1000848 (PMC2848560; doi:10.1371/journal.ppat.1000848)
Supplement: Table S1 — Oligonucleotides used for Southern analysis (0.03 MB DOC) [file ppat.1000848.s007.doc]

**Table S1. Oligonucleotides used for Southern analysis***

| Probe name | Chromosome location | Locus name | Forward primer | Reverse primer |
| --- | --- | --- | --- | --- |
| *chr1* | Chr1 | CNAG_07347 | AATGGGCTTTTGGGGTAAAC | CCTTGAGCGTTAGCCATTTC |
| *chr3* | Chr3 | CNAG_01580 | CTTGGCCATGATGTTGTTTG | CGACATTGCTCAAGTGGAGA |
| *chr4* | Chr4 | CNAG_05063 | AATGCAGCGCATACTCACAC | GCTTGGTGATGTCAAGCTCA |
| *chr10* | Chr10 | CNAG_04804 | GACCTCCGTCGTCTCAAAAA | AGGCCATGGGGGAGATATAC |
| *chr14* | Chr14 | CNAG_05465 | GCCGAGCACCTCATGTTC | TAAGCAACGACAGCCCAGAC |

*PCR product of each primer pair was used as probe for Southern blot analysis.
