# Supplementary material for: Cryptococcus neoformans Overcomes Stress of Azole Drugs by Formation of Disomy in Specific Multiple Chromosomes
Source: PLoS Pathog. 2010 Apr 1;6(4):e1000848. doi: 10.1371/journal.ppat.1000848 (PMC2848560; doi:10.1371/journal.ppat.1000848)
Supplement: Table S2 — Oligonucleotides used for qPCR assays (0.04 MB DOC) [file ppat.1000848.s008.doc]

**Table S2. Oligonucleotides used for qPCR assays**

| **Probe name** | **Chromosome location** | **Locus name** | **Primer forward** | **Probe** |
| --- | --- | --- | --- | --- |
| *chr1A* | Chr1 | CNAG_00040 | forward 5’-TGGCAAGACGCCAAAGTCT-3’  reverse 5’-GGCGGCAAATCCCTTTTC-3’ | AACCCTGCCCGATGGCACGA |
| *chr1B* | Chr1 | CNAG_07334 | forward 5’-TCGCCATCTACGCCTACGA-3’  reverse 5’-TGGCACGAGAGTGGCATCT-3’ | CTTATCGTCCGTATGCTGCGTTACCGC |
| *chr1C* | Chr1 | CNAG_00730 | forward 5’-AATCCCTTCTTGCTCGTACTCTTC-3’  reverse 5’-GCCACATCCACTTCCTCCAA-3’ | TGTCACAGCCCCTCCACCCACTTT |
| *chr1D* | Chr1 | CNAG_00815 | forward 5’-GCCGTTGTTTGTAATTTGGGAAT-3’  reverse 5’-CCTGCCTATCCTTCAAGATCCTAA-3’ | AGTTCGCTAGGCACCCAATGTTGCC |
| *chr3A* | Chr3 | CNAG_02959 | forward 5’-GAAGATGGCGAGCCTGACA-3’  reverse 5’-CACTTCGAGCCTTCTTCTTCATG-3’ | CACCCAGGAGGAGCTCGCCGA |
| *chr4A* | Chr4 | CNAG_07830 | forward 5’-CCTTCAACTTCGTCCAATAATTCC-3’  reverse 5’-GCGCAAAGCCGACATAGAA-3’ | CTCGCGCCGAAGCATCTCGC |
| *chr4B* | Chr4 | CNAG_05063 | forward 5’-CATCGGCGCCAATTCTGT-3’  reverse 5’-TGTACGGGCGAGCGTCTT-3’ | AGCTGGCCGACTTTGGTACCGCC |
| *chr5A* | Chr5 | CNAG_00869 | forward 5’-ATCTCTCGCCCAAGCTCTTG-3’  reverse 5’-GGCCACGTTAACGGCAAA-3’ | CTGCTGCTCTCCTCATCCTTGGCC |
| *chr10A* | Chr10 | CNAG_04804 | forward 5’-AACTTCCACTCCTGAGCAGCTT-3’  reverse 5’-TCCGAGGCCGTCAACTGT-3’ | CCACTACCGATATTCGTGCACCTGACG |
| *chr11A* | Chr11 | CNAG_07554 | forward 5’-GCGACGCAGAGATGGACAA-3’  reverse 5’-CTTGGGCCTTTGCTCGAA-3’ | CGCTTCTTCTTCCGTCATTCGTTCAAAGTT |
| *chr14A* | Chr14 | CNAG_05465 | forward 5’-TCTCTTGTCGACGACCTCCAA-3’  reverse 5’-GGACCAGGCGAGGGAAGT-3’ | CCTCTCCGACAAGGCCCGCA |
